# Social Story Intervention for Training Expected Behaviors among Preschool Children: A Systematic Review and Meta-Analysis

**DOI:** 10.3390/ijerph21070940

**Published:** 2024-07-19

**Authors:** Ni Zhou, Li Zhou, Cheuk Yu Teresa Ho, Colman McGrath, Hai Ming Wong

**Affiliations:** Faculty of Dentistry, The University of Hong Kong, Hong Kong SAR, China

**Keywords:** behavioral training, social story, oral health promotion, preschool children, pediatric

## Abstract

Promoting appropriate behaviors in early childhood is crucial for children’s future development. This systematic review aimed to explore the efficacy of social story (SS) intervention in teaching expected behaviors among preschool children. A structured search strategy was applied to five online electronic databases. The references were systematically screened in accordance with the PRISMA statements. Randomized or non-randomized controlled studies, as well as single-subject studies, in which SSs served as a behavioral training approach for children aged 2 to 6 years were included. Information related to study design, characteristics of the participants, target behaviors, and implementation of SS intervention was extracted. A meta-analysis was performed using the random-effects model, where similar outcomes were evaluated by similar intervention across multiple studies. Twenty-one studies were identified for qualitative analysis, while two studies formed the basis of the meta-analysis. SS interventions were employed to teach a variety of behaviors among typically developing children as well as those with various disabilities, such as autism, developmental delay, hearing impairments, attention deficit hyperactivity disorder, or other disabilities. The target behaviors included oral health practices, peer interaction, staying on-task, self-regulation, sleep habits, and controlling aggressive behavior during group activities. The SSs were used either alone or combined with other strategies, such as positive reinforcement, music therapy, role play, group discussion, video self-modeling, immediate practices, or additional audio commentary. Most studies reported improvements in appropriate behaviors and/or reductions in unfavorable behaviors. The meta-analysis indicated that children practiced more toothbrushing steps when using SS interventions compared to conventional oral health instruction (Z = 3.60, MD = 0.66, 95%CI 0.30 to 1.02, *p* < 0.001). SS interventions have the potential to teach target behaviors, particularly toothbrushing behaviors, among preschool children. More well-designed randomized controlled trials are warranted to determine the efficacy of SS interventions among children with various developmental profiles.

## 1. Introduction

Social stories (SSs) were initially introduced by Gray and Garand as an approach for teaching social skills to individuals with autism, since they exhibit impairments that impede their ability to understand social situations [1,2]. An SS typically contains brief passages of information, written within the child’s comprehension level, and assisted by visual depictions to deliver instructions about a specific social situation [3]. The rationale behind social stories was based on the ‘understanding of social cognition in autism’ [1]. However, increasing evidence supports that SS intervention can be generalized from social situations to daily life circumstances, as they can serve as narrative descriptions of daily events, elucidating what occurs and why in a clear and reassuring manner suitable for a child’s understanding [4,5,6]. Furthermore, the vocabulary employed in an SS tends to incorporate positive language, which aids in maintaining the audience’s focus on the target topic [5]. Over the past two decades, SSs have been widely used by teachers, parents, and other professionals to teach various kinds of skills or train the expected behaviors among individuals who lack the ability to understand social cues, including lunchtime eating behaviors, tooth-friendly eating habits, visiting a dentist, toothbrushing, sitting appropriately, on-task behavior, and communication skills [6,7,8,9].

Behaviors are associated with children’s emotional and physical well-being. Children who display deficits in social behaviors are reported to have difficulties in developing meaningful friendships with their counterparts [10]. Inappropriate sedentary behavior or eating habits can expose children to a higher risk of obesity, which can generate a negative impact on children’s psychosocial health [11]. Shaping children’s behavior patterns, such as reducing the frequency of between-meal snacking and establishing regular dental visits and tooth-brushing habits, can reduce the risk of tooth decay. Therefore, fostering appropriate behaviors (e.g., social interaction, dietary behaviors, personal hygiene behaviors) in early childhood can set a solid foundation for children’s future development.

As a behavior management approach, SS intervention causes no harm or stigma to the users, and it can be embedded in a variety of daily routines [12]. Marion et al. had used SSs to prepare children with autism for dental visits, and 64% of caregivers found the dental story useful for themselves and their child [13]. In addition, SSs can be intentionally tailored to meet the specific need of a particular learner, so that the reader can be engaged and motivated to practice the target behavior [14]. According to a questionnaire-based study, the majority of teachers (96%) reported that they always wrote SSs specifically for a particular student, creating personalized SSs for individual learners rather than using generic ones [15].

Although there is evidence that SSs have gained popularity among teachers or healthcare providers working with children, the efficacy of SS intervention for modifying behaviors among preschool children has rarely been synthesized. Several systematic reviews have been conducted to synthesize the application of SS interventions among children and adolescents. However, those systematic reviews primarily focused on individuals with autism, aged between 3 and 15 years, and only included single subjects or single case studies [7,16]. Another study conducted a qualitative analysis of six controlled trials, investigating the effectiveness of SS in enhancing social skills among children with autism, aged 4 to 14 years [17]. Considering that existing systematic reviews only included individuals with autism, it remains unclear whether the SS interventions can be generalized to a broader population. Additionally, these reviews mainly focused on social skills, leaving the question of whether SS interventions can be used to teach other behaviors unanswered. Therefore, we will systematically search for studies that utilize social stories to teach desired behaviors to preschool children, aiming to evaluate the efficacy of SS interventions in behavior training among preschool children, regardless of whether they have been diagnosed with autism or not.

## 2. Materials and Methods

### 2.1. Focused Question and Search Strategy

This systematic review was registered with PROSPERO (Registration ID: CRD42019115425). The research question of this study was defined as “When compared to conventional interventions or baseline data, will the target behaviors of preschool children be improved by using SS interventions?” Five online databases were retrieved, including PubMed, British Education Index, Web of Science, Scopus, and the Education Research Information Center (ERIC) from the establishment dates of these databases through to April 2021. The databases were recommended by an experienced librarian. Search terms related to ‘social story’ and ‘behavior training’ were mainly abstracted from published systematic reviews and meta-analyses [7,16,17]. The keywords in relation to ‘social story’ included ‘social stories’, ‘social story’, and ‘social story™’. Terms associated with ‘target behaviors’ encompassed ‘social initiations’, ‘social interactions’, ‘behaviors’, ‘skills’, ‘adaptive functioning’, ‘self-care’, ‘everyday activities’, ‘training’, ‘achievement’, ‘health education’, and ‘health promotion’ (Supplementary S1).

### 2.2. Study Selection and Selection Criteria

The selection process strictly adhered to PRISMA (Preferred Reporting Items for Systematic reviews and Meta-Analyses) statements [18]. The included studies had to meet the following pre-defined criteria: (i) study design: studies in which information regarding changes in target behaviors were reported before and after SS intervention, including randomized controlled trials (RCTs), non-randomized controlled trials, single-subject design, etc.; (ii) main intervention: SS-based intervention; (iii) participants: children aged between 2 and 6 years, with or without special care needs; (iv) language: English. The exclusion criteria included (i) observational studies without any SS interventions; (ii) participants below the age of 2 or over the age of 6; (iii) SS not being the primary intervention; (iv) target behaviors not being adequately defined; (v) full-text not being available; (vi) relevant data not able to be extracted. Upon removing duplicates from the five online databases, the titles and abstracts of the remaining records were screened by two investigators (NZ and HMW), aiming to identify studies that could potentially meet the inclusion criteria. Full texts of the studies identified in the first round were assessed in detail to confirm whether they met all the specified inclusion criteria. The ‘eligible’ studies were included for qualitative analysis, while studies with similar outcomes and similar interventions were included for quantitative analysis. Disagreement between the two reviewers was resolved through discussion with a third reviewer.

### 2.3. Quality Assessment of the Included Controlled Trials

Among the selective studies, bias assessment of controlled trials was analyzed in accordance with the “Cochrane Collaboration’s tool for assessing risk of bias” [19], which involved 6 domains, namely random sequence generation (selection bias), allocation concealment (selection bias), blinding of participants and personnel (performance bias), blinding of outcome assessment (detection bias), incomplete outcome data (attrition bias), selective reporting (reporting bias), and other bias (potential source of bias related to the specific study design in individual studies).

### 2.4. Data Extraction and Synthesis

A pre-defined table was developed to extract data from the selected studies, including authors, year of publication, study design, duration of the study, characteristics of the participants (developmental condition, age, sample size), target behaviors (behaviors which were expected to be modified after the intervention), and key findings (whether the target behaviors had improved or not). The development and implementation process of the SS intervention were also summarized in the pre-defined table. The principal findings of the included studies were analyzed by narrative synthesis. If similar behavioral outcomes were yielded by similar intervention in multiple studies, meta-analysis would be performed by using the random-effects model. The forest plot was generated by using RevMan Web (Version: 8.1.1). The effect size of continuous data was presented in the format of mean and standard deviation (SD), which were pooled from the primary studies.

## 3. Results

### 3.1. Characteristics of the Selected Studies

**Figure 1:**
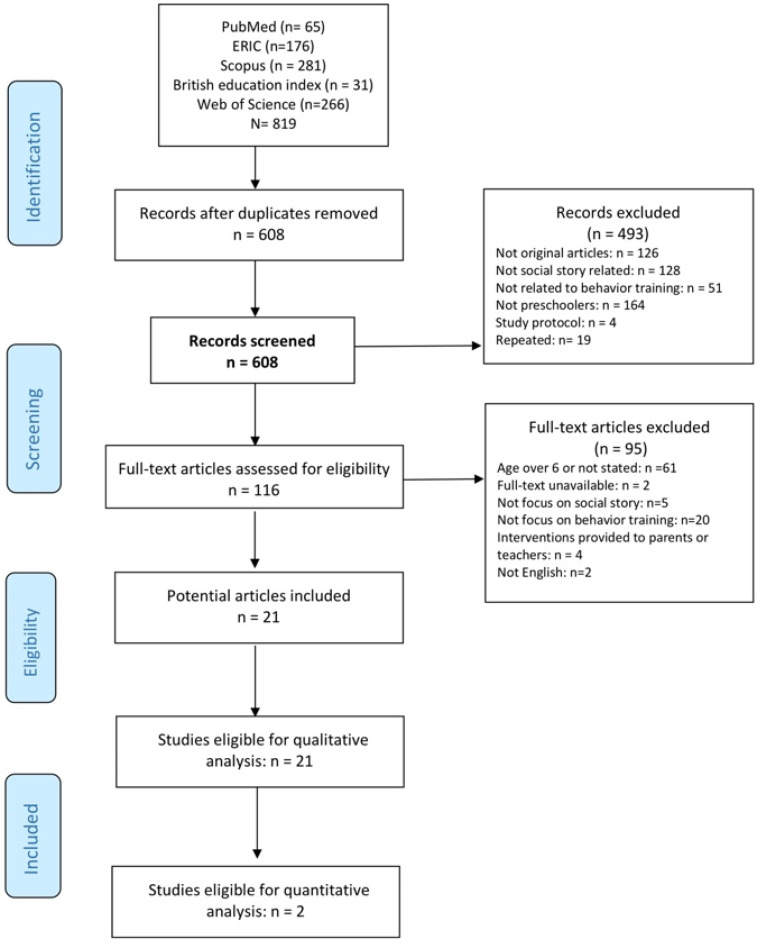
PRISMA flow diagram of study selection.

A total of 819 records had been retrieved from the five online electronic databases. After removal of the duplications, 608 records remained, and 116 potentially eligible studies were identified by the initial screening of titles and abstracts. After reading the full text, 21 articles met the inclusion criteria (Figure 1). The Kappa value of inter-rater agreement was 0.89.

**Figure 2:**
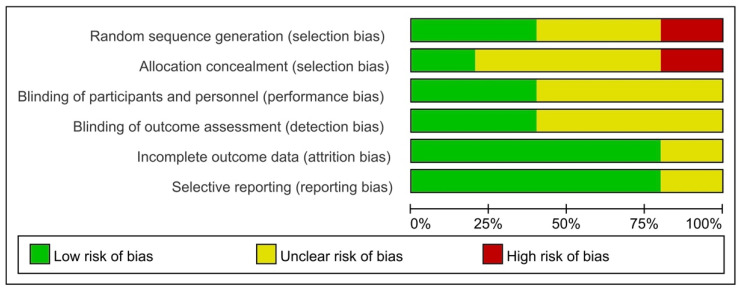
Risk of bias item presented as percentages across the controlled trials (n = 5).

The selected studies were mainly published within the timeframe of 2002 to 2021. These studies illustrated the changes in children’s target behaviors before and after the implementation of the SS intervention. A total of 16 (76.2%) studies used ‘single-subject design’, including ABAB design [20], ABA/ACABA design [21], ABCB design [22], ABCA design [23], reversal design [24], multi-probe design [25], multiple baselines across participants design [4,26,27,28], multiple baselines across behaviors design [2], ABC multiple-baseline design [29], adapted alternating treatment design [10], and pre-experimental [30] or AB design [3]. One study did not state the type of single-subject design [31]. Five studies had set up control groups [8,9,32,33,34]. Of those, one study [32] was designed as a two-arm parallel controlled trial, comparing the efficacy of SS intervention combined with or without additional practices. One study [33] assessed the behavioral changes between children who received SS intervention and their peers who received no intervention. Another study [34] compared the efficacy of SS intervention between children with special needs with and without autism. One study Two studies [8,9] compared the efficacy of SS intervention in oral health education with the conventional oral health instruction. High risk bias of ‘random sequence generation’ existed in one study [34], where children were allocated into two groups based on developmental profile instead of using a random allocation method. More than half of the included controlled trials showed ‘unclear risk’ of performance bias and detection bias. ‘Low risk’ of bias was evident among the controlled trials regarding attrition bias and reporting bias (Figure 2).

### 3.2. Characteristics of the Included Preschool Children

The selected studies involved 921 children aged 2 to 6 years. The sample size of each study ranged between 1 and 352. Sixteen studies recruited 1 to 3 participants, and only two studies had a relatively larger sample size over 300 [8,9]. Children with autism or Asperger syndrome were involved in most (17 out of 21 studies) of the eligible studies. Additionally, ‘typically developing’ preschoolers [29] and children with aggressive behaviors [33], maladaptive social behaviors [30], developmental delay [22,28], hearing impairments [3], attention deficit hyperactivity disorder, obsessive compulsive disorder, or other disabilities [8,28,34] were also recruited in the selected studies (Table 1).

### 3.3. Characteristics of the SS Interventions

The specific criteria or process for developing the SSs were not illustrated in 3 studies [3,20,33], whereas most (18 out of 21 studies) of the SSs were developed following the guidelines recommended by Gray. However, those guidelines were proposed in various timepoints, including the years 1993 [2,9,23], 1994 [10], 1995 [24,25,28,31], 2000 [21,22], 2002 [24,30], 2004 [8,27,29,32], and 2010 [4,8]. Additionally, in one study [31], the illustrations in the SSs were created based on child’s favorite cartoon characters, whereas in another study [30], the illustrations in their SSs were pictures of the target population when they were exhibiting the desired behaviors. The SSs which had been developed in the included studies were applied with various training purposes, including the following:

(i) Teaching oral health-related behaviors—toothbrushing skills [8,9,34], tooth-friendly eating habit [8], and dental visit process [8].
(ii) Teaching daily living skills—removing or spitting chewed food from mouth [21] and controlling challenging behaviors surrounding sleep and bedtimes [31].
(iii) Teaching peer interactions or social engagement—sharing toys [21,32], greeting [2], playing turn-taking [3], hand raising in a class setting [22,25], social engagement with peers or peer interaction [10,32], cultivating on-task behaviors [4], and reducing hitting or screaming during group activities [23,28].
(iv) Cultivating self-regulation behaviors or self-managed coping skills [27,30].
(v) Reducing tantrum behaviors [20] or aggressive behaviors [33].

One to five SSs were delivered to the preschool children in each study. The stories were mainly presented in book format, and those stories were read by parents [2,9,20,21,31,34], teachers [22,23,29,30,32], master’s students [3], therapists [20,27], or investigators [10] at home, in class, or in summer program settings. Additionally, two studies [4,26] applied digital device-assisted SS interventions to the recruited children, in which iPads and computers were employed to present the stories. Children could access the stories by tapping the screen, and the SSs were read by using a synthesized digital voice. One study [30] presented the SSs in the apron storyboard format. One study [28] transformed the SSs into SS songs, and children sang along while the SS songs were played by a CD player.

The follow-up periods of the SS interventions varied from 3 days to 24 months. The SSs used in the selected studies were delivered solely [20,22], or incorporated with other strategies, such as immediate practices [3,21,24,27], positive reinforcement [10,23,28,31], sensory integrative-based strategies [27], role play [25,33], video self-modeling [2], additional audio commentary [4], apron storyboard [30], music therapy [28], oral health instruction (OHI) or toothbrushing training [8,9,34], giving feedback [33], and discussion or asking questions [24,25,27,29]. SS interventions described in the selected studies were combined with one or multiple incorporated strategies. In addition, one controlled study compared the efficacy of SS-only intervention with the efficacy of SS-plus-practice session intervention [32].

### 3.4. Qualitative Analysis of the Efficacy of SS Intervention in Behavioral Training

Improvement of the appropriated behaviors and/or reduction in the unfavorable behaviors were supported in most (18 out of 21 studies) of the eligible studies, whereas 2 studies [10,32] questioned the efficacy of SS intervention in behavioral training, and 1 study [4] highlighted that SS intervention was effective for some children but not all the children. One study [32] compared the efficacy of SS-only intervention with the efficacy of SS-plus-practice session intervention, and found that sole SS intervention was not effective in behavioral training among preschool children. A total of 376 (40.8%) preschoolers with autism were recruited in 12 studies, and 10 studies supported the efficacy of SS intervention in modifying the target behaviors among preschool children with autism. One study [34] compared the efficacy of SS intervention between children with and without autism. The main findings suggested that the oral hygiene status and toothbrushing skills of children with and without autism were significantly improved by SS intervention, whereas children with autism showed better oral hygiene status than their peers without autism.

### 3.5. Quantitative Analysis of the Efficacy of SS Intervention in Behavioral Training

**Figure 3:**
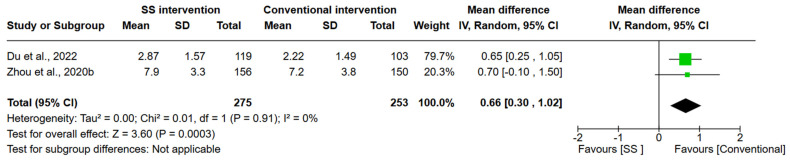
Mean difference in toothbrushing steps practiced by children who received SS intervention vs. convention intervention (Mean and SD pooled from 2 trials [8,9].

Two controlled studies [8,9] compared the efficacy of SS intervention in improving the toothbrushing behaviors with the conventional OHI, and both of them reported changes in toothbrushing steps before and after SS intervention. The outcome of those studies was toothbrushing performance measured by the number of toothbrushing steps. In one study [9], which initially involved 352 children, the ‘key toothbrushing steps’ at the 3-month and 6-month follow-ups were reported. There were significant differences in key steps between the test and control groups. The other study [8], which involved 306 children, assessed the number of toothbrushing steps at 6-month, 12-month, and 24-month follow-ups. There were no statistical differences in the toothbrushing steps between the two groups at 6-month follow-up, while significant differences were observed after 12 and 24 months. The random-effects model was adopted to synthesize the number of toothbrushing steps after 6-month SS intervention. The forest plot demonstrated that SS intervention was more efficient in improving the number of toothbrushing steps when comparing to the conventional intervention (Z = 3.60, MD = 0.66, 95%CI 0.30 to 1.02, *p* < 0.001, Figure 3).

## 4. Discussion

This systematic review and meta-analysis searched the relevant studies from five online databases (British Education Index, ERIC, PubMed, Scopus, and Web of Science). In the selected studies, SS interventions were implemented in various settings to teach various expected behaviors for the preschool children with or without special needs. Among those studies, SSs were mainly used among children with autism, and most of them supported the efficacy of SS intervention in training expected behaviors among the recruited children. Meanwhile, several studies demonstrate that SSs could be used to teach desired behaviors among children without autism. Benish and Bramlett (2011) stated that SS intervention could be used to teach peer interaction among typically developing preschool children [29]. Raver and colleagues (2013) reported that preschool children who were wearing hearing aids could achieve the target behaviors after using SS intervention [3]. Moreover, Gray and Garand (1993) claimed that SSs were most likely to benefit students functioning intellectually in the trainable mentally impaired range or higher who possessed basic language skills [1]. This indicated that the use of SSs could be generalized to a wider population.

SSs could be presented in the format of a booklet, songs, or on a digital device. Preschool children had limited comprehension levels to read a book independently. When a SS was delivered in the format of conventional booklets or brochures, teachers, parents, or investigators had to read the stories for those children [10,22,29]. Vandermeer and colleagues used iPads to present the SSs [4]. They suggested that digital devices could deliver SSs in a motivating and engaging way. By tapping the screen, children could see the illustrators and listen to the stories independently. However, to date, few randomized control trials have been conducted to compare the efficacy of SSs mediated by booklets and digital devices. It remains unclear which approach is more efficient to demonstrate an SS for young children.

In the selected studies, SSs had been employed to teach a wide array of behaviors for preschool children, and these stories could be implemented alone, or combined with other strategies. When implementing SS intervention, one of the key points was connecting the stories to the real situation, so that children could grasp the optimal timing to practice the target behaviors. In some studies, SSs were read prior to the situations in which the target behaviors would typically occur [21,24]. By doing this, children could immediately apply what they had learned from the SSs into real practices. If it was not feasible to predict when the target situation would occur, extra practice or role play could be arranged after a child had read the stories. It is well documented that when SSs are implemented along with immediate practices, an increase in the expected behaviors could be achieved by young children [3,21,24,25,27].

Another key point was enhancing children’s understanding of the SSs and strengthening their memories about the target behavior described in an SS. Young children might have limited literacy to grasp the essentials of the entire story. If the information conveyed by an SS had not been fully understood by the participants, the intervention efficacy would be compromised. Thompson and Johnston organized a group discussion after reading the stories [27], while other investigators included a section of “role play” or “questions and answers” in their studies [10,25,29]. Thus, teachers and parents could interpret the stories to their children on an individual basis. It was reported that children could learn new things by watching others, imitating actions, and receiving hints from subtle social cues [14]. When joining a group discussion or role play, the participants could share personal experiences with their counterparts and learn the target skills from each other. Furthermore, some studies used other approaches to make the participants more familiar with the target behaviors described in an SS. For example, after a teacher had read the stories, children were instructed to repeat the story immediately [32]. Another approach to refresh children’s memory about the target behaviors is by reading the SSs regularly, for instance, 2 to 3 times per week [3], 4 days a week [22,32], or at least 25 h per week [4]. When SS intervention had been implemented efficiently, the expected behaviors could continue to occur (maintenance phase), even after the intervention had been withdrawn [23,25].

One limitation of this systematic review was the small sample size in most of the selected studies, which might reduce the power to detect the true effect size of the SS intervention. This limitation has also been identified by More and colleagues, highlighting that previous studies on SS intervention mainly focused on single-subject or case study designs, and there has been limited research employing larger samples and/or experimental methodologies [32]. Another limitation was that a meta-analysis was not performed in single-subject design studies. This was due to the fact that there were different types of single-subject designs, including pre-experimental (AB) design, withdrawal (ABA/ABAB) designs, multiple-baseline/multiple-probe designs, multiple-treatment design, changing criteria designs, alternating treatment designs, adapted alternating treatments designs, etc. [35]. Among the included studies, the specific design of the single-subject studies was inconsistent. The target behaviors, outcome measures, intervention durations, timing for reading SSs, and delivery of SSs also varied across the eligible studies. Due to heterogeneity, it was not feasible to synthesize the outcomes of the single-subject studies using meta-analysis. Additionally, the criteria for developing SSs were inconsistent across the included studies. The majority of studies reported that their stories were developed in accordance with the guidelines proposed by Gray [2,4,10,21,22,23,24,25,27,29,32]. As Gray had updated her guidelines occasionally, the guidelines used by the included studies were released in different years. Although the above guidelines changed subtly through each revision, it remains unknown whether one set of guidelines is more efficacious than others [10,32]. Therefore, it is difficult to determine whether the inconsistent criteria for developing an SS could bias the main findings of the selected studies. Additionally, this review includes studies published up to 2021, and the most recent implementations of SS interventions among preschool children may not be reflected. Nonetheless, few recent studies have been published in this field. Despite these limitations, SS intervention has gained popularity in managing challenging behaviors among pediatric populations. More well-designed studies are anticipated to provide further insights into the effectiveness and application of SS interventions across diverse populations.

Given the evidence that children’s toothbrushing performance could be improved significantly following SS intervention, future studies are recommended to investigate the feasibility and effectiveness of SS interventions in oral health promotion across young pediatric populations. Additionally, with the increasing popularity of Generative Artificial Intelligence (GenAI) and the recommendation that digital devices can effectively deliver SSs to young children in a motivating and engaging manner [4,14,26], further studies are suggested to explore the application of GenAI tools in streamlining and enhancing the development of SSs. Moreover, comparisons of SS interventions with other behavioral training approaches, such as video modeling, comic strip conversations, or play therapy, are also encouraged. This could shed light on the most effective strategies for teaching expected behaviors among children.

## 5. Conclusions

SS interventions have been applied to teach appropriate behaviors among preschool children, and they are implemented across various settings. Those stories are mainly used among children with autism, whereas preschool children without special needs or children with other developmental problems may also benefit from SS intervention. SSs can be presented in various formats, such as booklets or songs or via digital devices, and can be used independently or in combination with other strategies. The efficacy of SS intervention in toothbrushing training was supported by trials, indicating that SS intervention was more efficient than the conventional instruction in improving the number of toothbrushing steps (Z = 3.60, MD = 0.66, 95%CI 0.30 to 1.02, *p* < 0.001) among preschool children with special care needs. To further validate these findings, more well-designed randomized controlled trials with larger sample sizes are required. These trials are recommended to facilitate comparisons between the effectiveness of SS interventions and other behavioral training approaches in modifying expected behaviors among preschool children with different developmental profiles.

## Figures and Tables

**Table 1:** Characteristics of the selected studies (*n* = 21).

Study	Design	Participant			Target Behaviors	Implementation of SS Intervention	Key Findings
		Condition	*n*	Age (y)			
Lorimer et al., 2002 [20]	Single-subject ABAB design	ASD	1	5	Tantrum behavior	Parent and therapist viewed Gray’s videotape presentation (1996) and read 2 SSs to child each morning at home, 3 weeks	Decrease in challenging behaviors when SS presented; increase in challenging behaviors after SS withdrawn
Kuoch & Mirenda 2003 [21]	Single-subject ABA/ACABA design	ASD	3	3–5	Sharing toys, playing with peers, hands in pants, making sounds, removing or spitting chewed food from mouth	Read directly prior to situations in which the target behaviors typically occurred, 2–4 min per session, 4 weeks	Low rate of problem behaviors was maintained. Irreversible learning of appropriate behaviors may have occurred during the course of the interventions
Agosta et al., 2004 [23]	Single-subject ABCA design	ASD	1	6	Screaming during group activities	Paired with reinforcement system, 35 days	Number of screams decreased; appropriate quiet behavior increased and maintained
Moore 2004 [31]	Single-subject design	ASD	1	4	Behavioral problems surrounding sleep and bedtimes	SS read with mother before bedtime, and implemented with a laminated reinforcement chart, monitored regularly by phone calls, 4 weeks	Sleeping behavior was modified after intervention
Haggerty et al., 2005 [30]	Single-subject pre-experimental design	Maladaptive social behaviors	1	6	Self-managed coping skills	SS + apron storyboard, teacher read the story to child, 4 weeks	Reduction in inappropriate behaviors after implementing the SS and apron storytelling
Crozier & Tincani 2007 [24]	Single-subject ‘reversal design’	ASD	3	3–5	Sitting, talking and playing with peers appropriately	Immediately prior to the target activity the SSs were read, 3 weeks	Reduction in inappropriate behaviors; increase in appropriate behaviors
Chan & O’Reilly 2008 [25]	Single-subject multiple-probe design	ASD	2	5–6	Hand raising, social initiations and vocalizations	Read, ask questions and role play, 10 months	Appropriate behaviors increased; inappropriate behaviors decreased; effects maintained for 10 m
Ozdemir 2008 [26]	Single-subject multiple-baseline across participants design	ASD	3	5–6	Appropriate social engagement behaviors	Multimedia SSs, 10 min play sessions, 3 times/week, 45 sessions	Some appropriate engagement increased after intervention
De Mers et al., 2009 [28]	Single-subject multiple-baseline across participants design	ADHD, DD, OCD, AS	3	5–6	Hitting, screaming (decrease), asking (increase)	Incorporated music therapy and reinforces (5 SS songs), 3 weeks	Decrease problem behaviors and increased alternative behaviors observed, and the effect continued 3 w after intervention
Litras et al., 2010 [2]	Single-subject multiple-baseline across behaviors design	ASD	1	3	Greeting, inviting to play, and contingent responding	Combined with VSM, delivered by parents, without prompts or reinforcement, 3 weeks	Communication and social engagement improved after intervention
Benish & Bramlett 2011 [29]	Single-subject ABC multiple-baseline across participant design	Normally developing	3	4	Peer interaction	Child-specific (target the specific needs of each participant); teacher read and asked questions, 3–5 days	Aggressive behavior decreased and positive social interactions increased
Hsu et al., 2012 [22]	Single-subject ABCB design	AS, DD	3	3–6	Hand raising; sitting in one’s seat; following teacher’s directions	Read by teachers, once daily, 4 days a week, 3 weeks	Expected behaviors increased after intervention
More et al., 2013 [32]	Parallel controlled trial	with and without disabilities	32	3–6	Joining in, sharing toys, asking to join a play group	Grp1: teacher read, student repeated; Grp2: teacher read, student repeated and practiced, 4 days per week, 9 weeks	Not effective
Raver et al., 2013 [3]	Single-subject AB design	Hearing impairment	2	4	Initiating verbal comments, responding to verbal comments, and play turn-taking	Read for 2–3 min; played for 5 min, 2–3 times/week, 16 weeks	The target behaviors described in the SSs were achieved after intervention
Thompson &Johnston 2013 [27]	Single-subject multiple-baseline across participants design	ASD	3	3–5	Self-regulation behaviors	Combined with sensory integrative-based strategies; read, discussed and practiced, 5–13 min/session, 9 weeks	Desired behaviors increased; incorporating other strategies into SS intervention was suggested
Kassardjian et al., 2014 [10]	Single-subject adapted alternating treatment design	ASD	3	5	Explaining a prior “cool” event to their peers	Researchers read, children who corresponded correctly were awarded, 3 months	Not effective
Vandermeer et al., 2015 [4]	Single-subject multiple-baseline across participants design	ASD	3	4	Appropriate and typical on-task behavior	Incorporated with photographs and audio commentary; child could tap screen and listen to story, ≥25 h per week, 40 weeks	The combination of the SS together with the iPad proved to be an effective intervention for one child (not all children)
Al Sayed 2018 [33]	“Semi experimental design”	Aggressive children	14	4–5	Aggressive behaviors	Grp 1: SS paired with instructions, modeling, role play and feedback; Grp 2: no intervention, 21 sessions	Children’s aggressive behaviors decreased after SS interventions.
Zhou et al., 2020a [34]	“two-arm, preintervention, and postintervention trial”	Special-needs children with and without ASD	181	2–6	Toothbrushing skills	A dental assistant showed parents how to use SS; parents read SS with children before/during toothbrushing Grp 1: children with ASD Grp 2: children without ASD 6 months	SS intervention was efficient in improving toothbrushing skills
Zhou et al., 2020b [8]	RCT	SHCN	306	2–6	Toothbrushing steps and frequencies, between-meal snacking frequencies, dental visit behaviors	A dental assistant demonstrated how to use the OHE materials; parents read to children at home Grp 1: SSs Grp 2: Conventional OHE leaflets 24 months	Oral health-related behaviors improved by using SS intervention
Du et al., 2022 [9]	Controlled trial	ASD	352	2–6	Toothbrushing skills	Grp1: Parents/teachers trained to read SS with children during toothbrushing Grp 2: conventional OHI 6 months	Significant improvements in toothbrushing skills

N: the number of recruited children; ADHD: attention deficit hyperactivity disorder; AS: Asperger’s syndrome; DD: developmental delay; Grp, group; OCD, obsessive compulsive disorder; OHE: oral health education; OHI: oral hygiene instruction; ID: intellectual disability; RCT: randomized controlled trial; VSM: video self-modeling.

## Data Availability

The data pooled from the original studies and/or analyzed during the present review are available from the corresponding author upon reasonable request.

## Supplementary Materials

The following supporting information can be downloaded at https://www.mdpi.com/article/10.3390/ijerph21070940/s1: S1: Full search strategy.
