# Supplementary material for: Social Story Intervention for Training Expected Behaviors among Preschool Children: A Systematic Review and Meta-Analysis
Source: Int J Environ Res Public Health. 2024 Jul 19;21(7):940. doi: 10.3390/ijerph21070940 (PMC11277040; doi:10.3390/ijerph21070940)
Supplement: Supplementary file 1 [file ijerph-21-00940-s001.zip › ijerph-3072286-supplementary.pdf]

Supplementary S1. Search Strategy

1.Pubmed

Terms related to social stories: (Social stories[Title/Abstract] OR social story[Title/Abstract]

Terms related to expected behaviours: initiations OR interactions OR training OR behavior OR behavious OR behaviors OR behavior OR communication OR skills OR adaptive OR achievement OR functioning OR self-care abilities OR everyday activities OR health promotion OR health education

2. Eric

("social stories" [Abstract] OR "social story" [Abstract]) AND (Initiations OR interactions OR training OR behavior* OR behaviour* OR communication OR skill* OR adaptive OR achievement OR functioning OR "self-care abilit*" OR "everyday activities" OR "health promotion" OR "health education")

3. Scopus

("social stories" [Abstract] OR "social story" [Abstract]) AND (Initiations OR interactions OR training OR behavior* OR behaviour* OR communication OR skill* OR adaptive OR achievement OR functioning OR "self-care abilit*" OR "everyday activities" OR "health promotion" OR "health education")

4. British education index

("social stories" [Abstract] OR "social story" [Abstract]) AND (Initiations OR interactions OR training OR behavior* OR behaviour* OR communication OR skill* OR adaptive OR achievement OR functioning OR "self-care abilit*" OR "everyday activities" OR "health promotion" OR "health education")

5.Web of science

("social story" OR "social stories") AND Subject: (Initiations OR interactions OR training OR behavior* OR behaviour* OR communication OR skill* OR adaptive OR achievement OR functioning OR "self-care abilit*" OR "everyday activities" OR "health promotion" OR "health education")
